# Writers Are Common among Parkinson's Disease Patients: A Longitudinal Study

**DOI:** 10.1155/2021/5559926

**Published:** 2021-05-06

**Authors:** Anna Aasly, Jan O. Aasly

**Affiliations:** ^1^Department of Neuromedicine and Movement Science (INB), Faculty of Medicine and Health Sciences, Norwegian University of Science and Technology (NTNU), Norway; ^2^Department of Neurology, St. Olavs Hospital, 7030 Trondheim, Norway

## Abstract

Parkinson's disease (PD) patients may have a specific personality profile, which includes being introvert, cautious and devoted to hard work. The evaluation of psychological characteristics must be evaluated according to methods for assessments of personality disorders. Such evaluations are often time-consuming and available only in research settings. The “parkinsonian trait” may be established early in life but may change with disease progression. To overcome this long interval before onset of PD questions on literary activities were included in the medical record. Three percent of PD patients could be defined as writers, significantly higher than observed in the general population. PD writers published their first books long before onset of disease. Being a writer is an extrovert trait meaning that the patient is prepared for criticism and publicity. We suggest that questions regarding personal activities prior to disease onset add valuable information on personality which differs significantly from traits observed later in the disease period.

## 1. Introduction

It has been claimed that patients with Parkinson's disease (PD) have a distinct personality and that there is a certain parkinsonian trait [1]. An experienced neurologist treating PD patients knows that this is generally a group of people who always show up on time; they are well-educated nonsmokers and with high degree of compliance. There have been a lot of studies which have tried to characterize the premorbid parkinsonian personality with diverse outcomes. The PD patient has been described as rigid, introverted, and cautious [2]. Robust and time-consuming methods are needed to evaluate personality profiles and psychiatric diseases through structured clinical interviews. This must be done in small cohorts at certain periods of the disease. It may give an accurate diagnosis of the individual's situation some years into the disease but will not always reflect the psychiatric and psychological conditions many years prior to the disease onset. Few if any did perform a personality test of future parkinsonian patients many years before the onset. This is critical since many studies have shown that cognitive decline is seen in a considerable proportion of patients at the time when they are diagnosed with PD, and this may definitely influence the personality test results.

The aim of this study was to use standardized anamnestic questions to evaluate personality factors in PD patients.

## 2. Methods

At outpatient visits, the PD patients seen by JAa were asked about their daily activities, including hobbies and employment. Over a period of 10 years, from 2010 through 2019, this also included a question regarding literary activities. There are probably many definitions of a writer. We used the International Standard Book Number (ISBN). To be a full writer, the patient's name had to be the name behind an ISBN-marked production. Most patients were asked: “Have you ever written a book?” There were patients who did not get that question mainly due to the doctor's prejudice.

This study was approved by the Ethics Committee of Central Norway, and informed consent was obtained from all participants.

## 3. Results

Forty-three patients confirmed that they had a literary activity above normal and had contributed substantially in writing one or more books. That makes 43/1315 = 3.3% of the PD cohort seen by one neurologist. Thirty-eight had written books by themselves without the assistance of other collaborators. Five patients, all were university professors, had written one or more book chapters in textbooks.

The writers and the nonwriters had similar age at the PD onset (Table 1) around 60 years, but the writers had significantly longer education and less smoking. Academic professions dominated in the group of writers, but there were big variations.

## 4. Discussion

This study was focused on the patient's activities reaching out to the community and was not a list of academic merits. Every year, there are about 10,000 books published in Norway, books translated from other languages not included, that is, an incidence of writers being equivalent to 0.3% of the adult population. About one-third of the publications coming to book stores are novels, fiction, poetry, prose, and imaginative literature. One-third is social science and application-required science. The rest was books within arts, geography, religion, and philosophy. A considerable percentage of writers are reappearing on the list of books from one year to the next.

The writers in our PD cohort reflected the publication activities well and were comparable to those reported by the national bureau of statistics. There were some who published novels, and others were concentrated on the expert knowledge within science or education. Some wrote books on local history, and others wrote poems. Like all other writers, the publishing activity varied. Fiction writers had published a book every or every other year. One expert within natural sciences had published more than 50 books, and another one had written more than twenty-five books on local history. Very few within this group of writers earned their own living from their writing activities. They were teachers or farmers, one was a sea captain, another was a salesman, or they were university employees [3]. But the significantly higher level of education among writers also confirms that high cognitive functioning is associated with PD [4].

Many patients were active within unions and committees, and as part of their positions, they had to write pamphlets and comments, but these were not included as writers. A lot of people like to write songs to birthday parties or sketches to students' shows. This may be a good training but did not qualify as being called a writer. Individuals involved in the writing of a master's degree and Ph.D. dissertation or scholarly articles are also not considered writers.

It is very difficult to assess the incidence and prevalence of true writers. In this cohort, some writers had a high production and others produced one book only, once in a lifetime. It is also difficult to give a good definition of a writer, but in general, it is a person who exposes oneself to others and must be prepared for feedback and criticism. It is not the type of activity one would expect from a person characterized as introverted, rigid, and cautious. All personality tests in PD patients must necessarily have to be done several years into the disease process. There is always some patient's delay before the diagnosis is verified, and it is usually some more delay before the test can be done and PET studies have shown that the disease process has started long before the patients' motor signs develop [5]. Although the personality profile is shaped early in life, it may probably drift to a more negative side when patients develop a chronic neurodegenerative disease. Information about the literary activities in the most active periods of an individual's period of life is probably a simple and predictive sign on personality. It is much more complicated to draw personality than to draw homologous conclusions from studying motor complications and freezing of gait many years into the disease process [6].

Many studies have shown that a considerably part of the patients have developed cognitive deficits at the time when they are diagnosed with PD which may influence the test results [7].

Depression is often included in the premorbid parkinsonian trait. It is so common that depression could be included a one of the cardinal signs of PD [8]. But depression is not a personality factor to be included in DSM V, axis 2; depression is a psychiatric disease which is diagnosed on axis 1. Every person has personality of some kind but not always a psychiatric disease.

We found that writers were more educated measured by years in school. This reflects the high number of books within professional and scientific literature. But many years in school is not a presumption for becoming an author. The Nobel Prize winner in literature in 1920, Knut Hamsun, had very little schooling, only 252 days in 6 years. The most ideal study of the premorbid parkinsonian personality should therefore be performed many years prior to the disease onset. Since the majority were not professional writers but had diverse backgrounds, this activity reflects the personalities of several groups of patients. In our study, most of the patients wrote their first book many years before they got sick. The development of a chronic disease did not stop the vein of poetry, and many continued their writing after the disease onset. The mean number of books written was *n*. But for many patients, the actual number of manuscripts was *n* + 0.5; the last manuscript became only half finished and was never published as PD dementia progressed.

## Figures and Tables

**Table 1:** Number of patients and writers.

Patients	No.	Male (%)	Age at onset	Years of education	Smoking^∗^
Nonwriters	1272	805 (63%)	59.5	11.2	4.8
Writers	44	36 (82%)	60.4	17.4	1.8

^∗^Smoking measured as sum of packs of cigarette/day and years.

## Data Availability

The data used to support the findings of this study are available from the corresponding author upon request.
